# FuSpot: a web-based tool for visual evaluation of fusion candidates

**DOI:** 10.1186/s12864-018-4486-3

**Published:** 2018-02-13

**Authors:** Jackson A. Killian, Taha M. Topiwala, Alex R. Pelletier, David E. Frankhouser, Pearlly S. Yan, Ralf Bundschuh

**Affiliations:** 1 Department of Physics, The Ohio State University, Columbus, OH USA; 2 Comprehensive Cancer Center, The Ohio State University, Columbus, OH USA; 3 Biomedical Sciences Graduate Program, The Ohio State University, Columbus, OH USA; 4 Division of Hematology, Department of Internal Medicine, The Ohio State University, Columbus, OH USA; 5 Department of Chemistry and Biochemistry, Center for RNA Biology, The Ohio State University, Columbus, OH USA

**Keywords:** Gene fusion, Visualization, RNA-Seq, Smith-waterman, Web tool, Fusion validation

## Abstract

**Background:**

Gene fusions often occur in cancer cells and in some cases are the main driver of oncogenesis. Correct identification of oncogenic gene fusions thus has implications for targeted cancer therapy. Recognition of this potential has led to the development of a myriad of sequencing-based fusion detection tools. However, given the same input, many of these detectors will find different fusion points or claim different sets of supporting data. Furthermore, the rate at which these tools falsely detect fusion events in data varies greatly. This discrepancy between tools underscores the fact that computation algorithms still cannot perfectly evaluate evidence; especially when provided with small amounts of supporting data as is typical in fusion detection. We assert that when evidence is provided in an easily digestible form, humans are more proficient in identifying true positives from false positives.

**Results:**

We have developed a web tool that, given the genomic coordinates of a candidate fusion breakpoint, will extract fusion and non-fusion reads adjacent to the fusion point from partner transcripts, and color code reads by transcript origin and read orientation for ease of intuitive inspection by the user. Fusion partner transcript read alignments are performed using a novel variant of the Smith-Waterman algorithm.

**Conclusions:**

Combined with dynamic filtering parameters, the visualization provided by our tool introduces a powerful new investigative step that allows researchers to comprehensively evaluate fusion evidence. Additionally, this allows quick identification of false positives that may deceive most fusion detectors, thus eliminating unnecessary gene fusion validation. We apply our visualization tool to publicly available datasets and provide examples of true as well as false positives reported by open source fusion detection tools.

## Background

Chromosomal translocations occur naturally in a wide variety of species from plants to mammals [1, 2]. The proteins resulting from fusion genes can be benign or even support the normal physiology of the organism [1]. However, in humans, gene fusions can also play an important role in carcinogenesis and the progression of cancer. This connection between genetic abnormalities and cancer was hypothesized as early as 1914 [3] and was confirmed with the discovery of the Philadelphia Chromosome—a miniscule, hybrid chromosome generated from a fusion between two chromosomes that was found exclusively in patients with certain leukemias [4, 5]. As a result of studying the tumorigenic role of an enzyme that this hybrid chromosome produced (tyrosine kinase), Druker et al. [6] were able to adapt a drug which inhibited the production of this enzyme (Imatinib), to become an effective oral treatment for some leukemias. This breakthrough highlights the targetable nature of oncogenic gene fusions thereby providing another avenue of cancer treatment in addition to standard chemotherapy. Furthermore, with the proliferation of deep sequencing techniques, studies have found that gene fusions occur in all major cancer subtypes [7]. This highlights the need and urgency in refining computational approaches that specialize in their detection.

With this surge of research interest, many computational tools have been developed for detecting known and novel fusion breakpoints in cancer genomes and transcriptomes. In several recent comprehensive reviews [8–10], the authors concluded that current detection tools are imperfect on their own as the efficacy of each tool is data dependent. There exists a trade-off between sensitivity and accuracy that has not yet been optimized by existing tools to accurately report all true positives with a negligible number of false positives. Therefore, when tested on the same data set, many detectors find or overlook different fusion points deduced from different sets of supporting data with varying degrees of accuracy. This observation was further discussed in a publication involving a synthetic fusion messenger Ribonucleic Acid Sequencing (mRNA-Seq) data set [11].

This discrepancy between tools underscores the fact that no computational method can perfectly evaluate evidence; especially when provided with small amounts of supporting data as is typical in fusion detection. In addition, since there are no consistent formats for tools to report their fusion candidates or metrics to report the associated levels of confidence, users must rely heavily on lab techniques to validate large numbers of putative fusion events, which is inefficient. Here we assert that if data is provided in an easily digestible form, the human eye can be used as a powerful tool to discern between true positives and false positives thereby eliminating time spent on validating false candidates. We have thus developed a tool that locally aligns and maps reads against fusion reference sequences to allow users to visualize and pinpoint fusion breakpoint evidence. Below we explain the algorithms behind the novel local alignment mechanism driving our tool and apply it to the output of selected fusion detectors that were run on publicly available data sets.

## Implementation

FuSpot is designed to allow users to critically inspect candidate fusion breakpoints derived from ribonucleic acid (RNA) fusion detector tools. This is accomplished by realigning reads adjacent to the breakpoint across references representing the breakpoint of interest to reveal alignment characteristics congruent with true or false gene fusions. FuSpot takes as input one file containing reads adjacent to the fusion point (two files for paired-end format). To create this input, FuSpot provides a tool that will extract from alignment files all reads and their mates adjacent to the fusion point. Once candidate fusion reads are gathered, to begin alignment the user will need to enter: 1) two genomic coordinates and strand information corresponding to the fusion breakpoint and 2) a base-pair distance used as a search radius centered at the breakpoint. From this, FuSpot will retrieve both genomic and exonic sequences flanking the breakpoint to use as an alignment reference. Alternatively, if the user prefers, they may upload custom reference files for FuSpot to use during the alignment step. The basis of the tool is a local alignment algorithm that incorporates a breakpoint and allows any number of references on the left side and the right side of the breakpoint. The process is based on a variation of the Smith-Waterman local alignment algorithm which generates a score matrix to calculate read alignments. The alignment algorithm allows for any number of insertions and deletions, allowing it to perform flexible alignments about junctions with various transcripts, as is frequent in fusion events.

### Gathering references around a fusion breakpoint

**Fig. 1:**
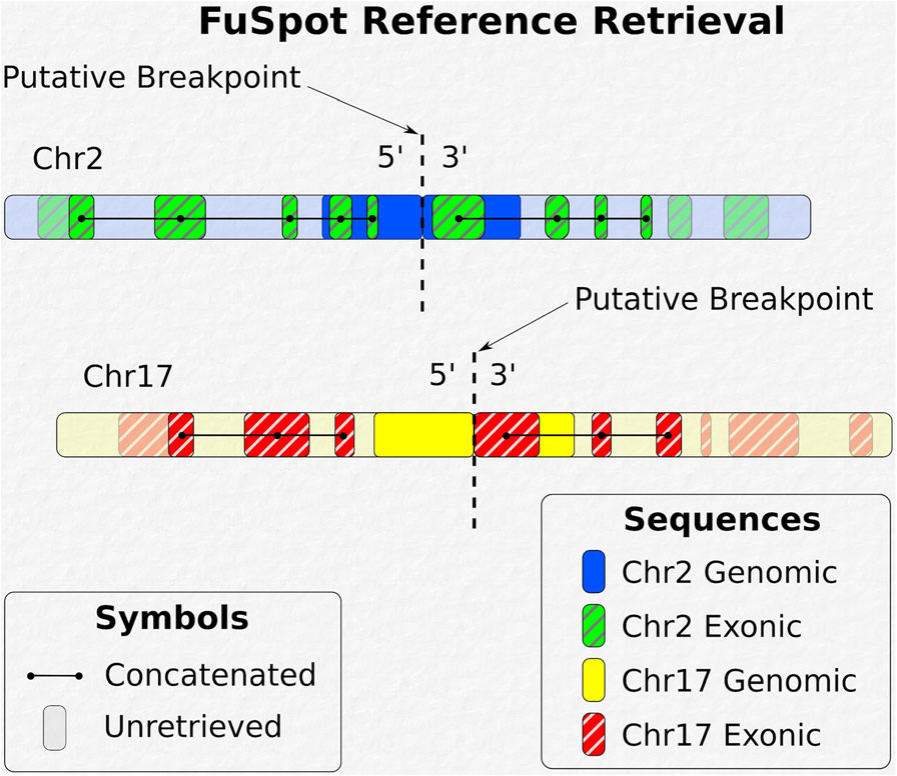
FuSpot Reference Sequence Retrieval. FuSpot reference retrieval is visualized. In this example, the putative fusion breakpoint exists between the rightmost base of the 5′ side of the Chr2 reference and the leftmost base of the 3′ side of the Chr17 reference. In order to perform alignments of reads which may align over the putative breakpoint or reads which may align to the normal sequence of either Chr2 or Chr17, FuSpot will gather references by collecting sequences of a defined length away from the breakpoint for both fusion gene partners. FuSpot will collect genomic sequences (always sequential — blue and yellow) and exonic sequences (potentially fragmented — green and red). Thus a total of 8 reference sequences are retrieved — 4 on either side of the breakpoint

FuSpot is built to perform alignments to fusion and non-fusion gene constructs simultaneously. To accomplish this, FuSpot will gather as references genomic sequences and exonic sequences flanking either side of the putative breakpoint for both fusion gene partners. References from both the genome and transcriptome are required to perform comprehensive alignments and visualizations since most fusion studies leverage RNA data. This sequence retrieval is depicted in Fig. 1.

To obtain the flanking genomic sequences for a user-specified fusion breakpoint, FuSpot will initiate a query to the University of California, Santa Cruz (UCSC) direct attached storage (DAS) server [12]. For a given side of the breakpoint, the base representing the coordinate of that side of the breakpoint will be depicted in the reference list matching the orientation of the fusion gene. If the reverse strand is specified for a given partner of the breakpoint, FuSpot will search in the 5′ direction for 3′ side references and vice versa, then finish by reversing and complementing the sequences. In the example below, if the input fusion breakpoint coordinates are between chr1:10,000:+ ➔ chr2:20,000:- and the user requests a 100 base pair (bp) search radius, the genomic references to be retrieved would be as follows:

**Left side genomic references:**

chr1:9,901–10,000

chr2:20,001–20,100

**Right side genomic references:**

chr1:10,001–10,100

chr2:19,901–20,000

where the sequences for chr2 are reverse complemented.

To retrieve exonic sequences, FuSpot follows this same stranded search convention and references a list of all exons obtained from the UCSC table browser [13] in order to query only the exonic regions of the genome. First, FuSpot checks if the breakpoint coordinate lies within the boundaries of an exon. If this is not true, it will then proceed to identify the boundary of the nearest exon in the direction of search to begin the query. Otherwise, the starting query position will follow the same convention described above. FuSpot will then calculate the distance from the starting position to the next boundary of the exon in the direction of search. If this distance is greater than the user-defined search radius, FuSpot will query the UCSC DAS server for the sequence between the starting point plus the distance of the search radius. However, if this distance is less than the user-defined search radius, FuSpot will query the DAS server for the sequence within this distance, then identify the boundary of the next nearest exon and mark that boundary as a starting point from which to search for subsequent exonic bases. Once FuSpot gathers enough exonic sequence fragments for the aggregate length to match the user-defined search radius, it will concatenate them in an order that maintains the strandedness with respect to the genome. Further, if the given breakpoint coordinate is specified in the reverse strand, the final concatenated sequence will be reverse complemented. For the example coordinates shown above, if a 200 bp exon existed at chr1:9801–10,000 and two 70 bp exons existed at chr1:10,031–10,100 and chr1:10,201–10,270, the exonic references associated with the first coordinate breakpoint would be as follows:

**Left side exonic references:**

chr1:9901–10,000.

**Right side exonic references:**

chr1:10,031–10,100, chr1:10,201–10,230.

FuSpot will extract references automatically as described above for the hg19 and GRCh38 builds of the human genome and the mm10 build of the mouse genome. In addition, FuSpot also allows users to upload their own sequences as references, allowing for an arbitrary number of references from any organism to be used as references on either side of the breakpoint. In this way, FuSpot builds in forward flexibility to accommodate fusion visualization from new genome builds and available organisms.

### Tool for extracting reads

Since current sequencing runs yield millions of reads, files detailing the alignments of all reads can be on the order of gigabytes. Since only a small portion of these reads is needed to evaluate each putative fusion breakpoint, and since files of such size are not suitable for upload to a web tool, we provide an extraction tool to collect relevant reads to be uploaded as input to FuSpot. This read gathering tool is available for download on the FuSpot website and has to be run locally by the user. Given the same search radius and breakpoint coordinates input to the reference gathering step, the read gathering tool will use PySAM [14, 15] to extract from input Binary Alignment Map (BAM) alignment files reads from all exonic regions out to an exonic distance matching the search radius away from the breakpoints. Additionally, it will gather all reads with alignments that start within a genomic distance equal to the search radius away from the breakpoints. If desired, paired mates for these reads will also be extracted regardless of their alignment. Exonic distances are calculated using the method described in *Gathering References Around A Fusion Breakpoint.*

In order to generate useful chimeric alignments, we recommend aligning reads with a chimeric-capable RNA aligner such as Spliced Transcripts Alignment to a Reference (STAR) [16]. Do note that when run in the chimeric mode, STAR will include some reads in both the normal alignment file and the chimeric alignment file. Therefore, if the read extraction tool is used to gather reads from both the chimeric and normal alignment BAM files, some reads may be doubly counted.

### Review of traditional Smith-Waterman alignment

Once the input reads and references are determined and submitted to FuSpot, alignment will commence. Since FuSpot’s alignment algorithm is a variant of Smith-Waterman local alignment, we will briefly review the Smith-Waterman algorithm as published by Temple F. Smith and Michael S. Waterman in 1981 [17].

### Score matrices

We will consider alignment of a read sequence A of length *m* and a reference sequence B of length *n*, whose bases are represented by *a*_*i*_, *b*_*j*_ respectively. A similarity function *σ* and a gap scoring scheme *W* must be defined to compare the bases of A and B. FuSpot declares these functions as follows:

$$ \sigma \left({a}_i,{b}_j\right)=\left\{\begin{array}{cc}1& {a}_i={b}_j\\ {}-1& {a}_i\ne {b}_j\end{array}\right\} $$

$$ W=-1. $$

The Smith-Waterman algorithm uses a matrix of scores for partial alignments of the sequences up to a specific pair of indices *(i,j)*. This Smith-Waterman score matrix *H* is generated as follows:

Initialization:

$$ H\left(i,0\right)=0,\kern0.75em 0\le i\le m $$

$$ H\left(0,j\right)=0,\kern0.75em 0\le j\le n $$

Scoring:

$$ H\left(i,j\right)=\max \left\{\begin{array}{c}\begin{array}{cc}0&\ \\ {}H\left(i-1,j-1\right)+\sigma \left({a}_i,{b}_j\right)& Match\ or\ Mismatch\end{array}\\ {}\begin{array}{cc}H\left(i,j-1\right)+W& \kern3.25em Deletion\ \\ {}H\left(i-1,j\right)+W& \kern3.5em Insertion\ \end{array}\end{array}\right\},{\displaystyle \begin{array}{c}\ 1\le i\le m,\\ {}1\le j\le n\end{array}} $$

Where *H*(*i*, *j*) is the matrix element of the similarity matrix.

**Fig. 2:**
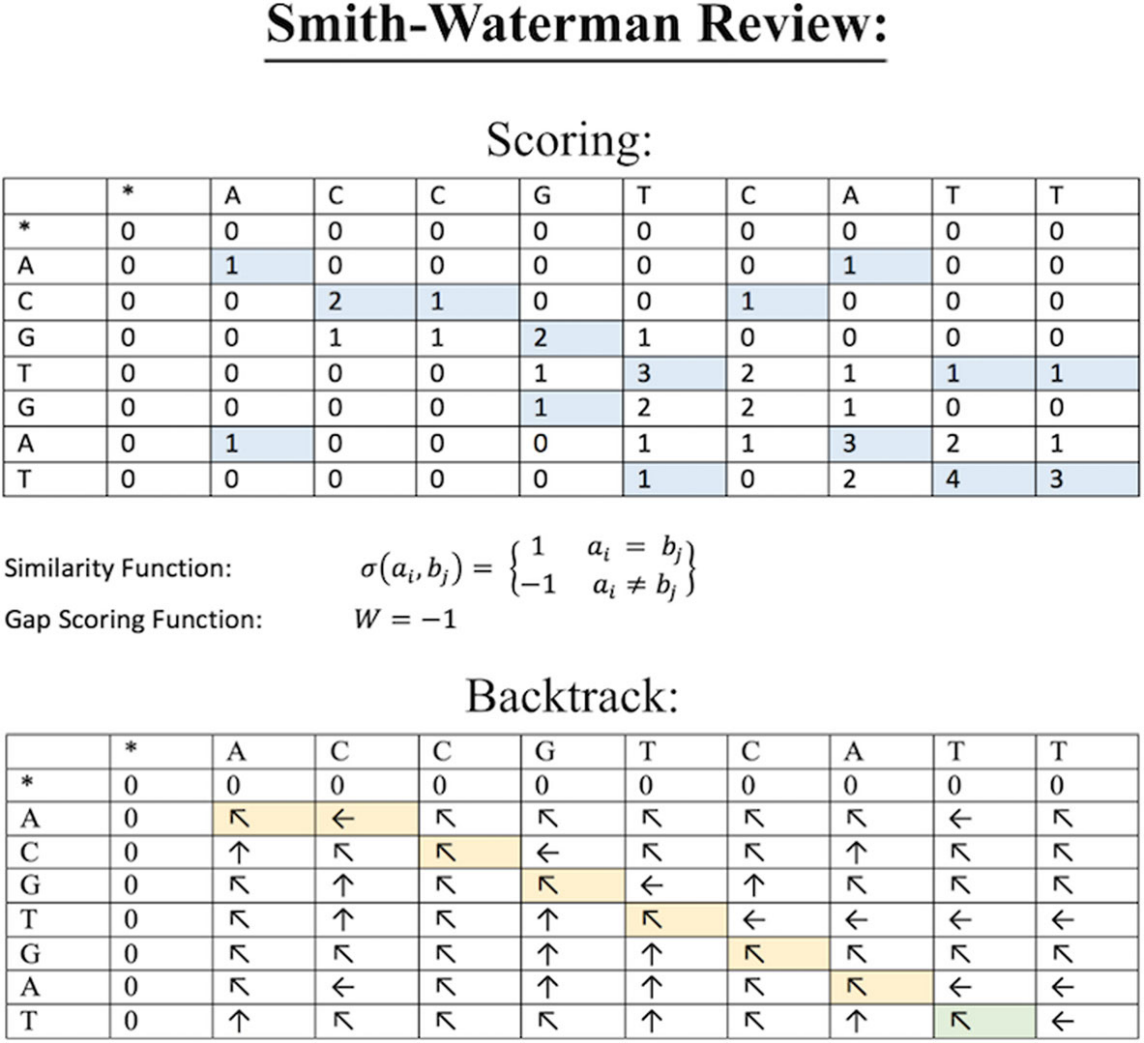
Smith Waterman Alignment Algorithm. (Scoring) Smith-Waterman alignment score matrix for the example read sequence, A, (given in the left-most column) against an example reference sequence, B (given in the top-most row) with the indicated scoring scheme. Each entry of the first row and first column are initialized to 0. Then each subsequent matrix element is calculated by taking of the maximum of the following three values: (1) Value of immediate left entry plus Gap Scoring Function, (2) Value of immediate top entry plus Gap Scoring Function, (3) Value of immediate top-left diagonal entry plus Similarity Function. (Backtrack) A tracing of the backtrack mechanism resultant from the above score matrix. Backtracking begins at the maximum matrix element (green) and continues to the matrix element from which its score was derived as defined by the scoring scheme. Movements to the diagonal produce a match or mismatch, movements to the top produce an insertion in the read, and movements to the left indicate a deletion in the read. This backtrack produces the alignment: Read: A _ C G T G A T; Reference: A C C G T C A T

This scoring process is visualized in Fig. 2.

### Backtrack and alignment

Once the matrix is populated, the alignment producing maximum similarity of the two sequences can be obtained by finding the maximum matrix element, then tracing it to its neighboring elements in descending order of element score:

$$ \left({i}_0,{j}_0\right)=\left(i,j\right)\kern0.3em where\kern0.3em H\left(i,j\right)=\max \left\{H\left({i}_y,{j}_z\right)\right\},\kern0.5em 1\le {i}_y\le m,1\le {j}_z\le n $$

$$ \left({i}_{q+1},{j}_{q+1}\right)=\left(i,j\right)\  where\ H\left(i,j\right)=\max \left\{\begin{array}{cc}H\left({i}_q-1,{j}_q-1\right)+\sigma \left({a}_i,{b}_j\right)& Diagonal\\ {}H\left({i}_q,{j}_q-1\right)+W& Left\\ {}H\left({i}_q-1,{j}_q\right)+W& Above\end{array}\kern0.75em \begin{array}{c}{A}_{out}+={a}_{i_q},{B}_{out}+={b}_{j_q}\ \\ {}{A}_{out}+={}^{"}-{}^{"},{B}_{out}+={b}_{j_q}\\ {}{A}_{out}+={a}_{i_q},{B}_{out}+={}^{"}-{}^{"}\kern0.5em \end{array}\right\}, until\kern0.5em {H}_q\left(i,j\right)=0 $$

Where: *A*_*out*_, *B*_*out*_ is the character sequence of the best alignment of A against B and B against A respectively.

This backtrack process is visualized in Fig. 2.

### FuSpot fanned alignment

In order to align reads from the vicinity of a fusion point, multiple references have to be available on both sides of the fusion point. At a minimum, reads on the 5′ side of the fusion point can be derived from the 5′ end of the messenger RNAs (mRNAs) of either of the two genes involved in the fusion. The same is true of the 3′ ends on the 3′ side of the fusion point, respectively. Alignments have to be able to “start” in either of the two possible 5′ ends and “end” in either of the two possible 3′ ends in order to identify the most parsimonious explanation for every read (purely gene 1, purely gene 2, or fusion of gene 1 and gene 2). Since mRNA-Seq data often also contains some amount of reads derived from unspliced precursor mRNA, it is also possible to obtain reads that represent the genomic sequences on either side of the fusion point from each of the two genes. Alternative splicing near the fusion point in either of the two genes would require even more possible references on one or both sides of the fusion point. Thus, the key feature of the alignment algorithm underlying FuSpot is that it allows a read to start in one of an arbitrary number of 5′ references, and continue through the common fusion point to one of an arbitrary number of 3′ references.

FuSpot’s algorithm introduces a third dimension to the Smith-Waterman algorithm by generating a single score matrix for each input reference and forcing them to converge and diverge at the fusion breakpoint. Effectively, FuSpot creates both a stack of score matrices corresponding to the left side references and a stack corresponding to the right side references; each side fans out from a center breakpoint column. These stacks of Smith-Waterman matrices will be represented as follows:

$$ {H}_s\left(i,j;f\right) $$

Where *S* will mark the side of the breakpoint column on which the stack lies and *f* will indicate the index of the score matrix and corresponding reference on that side.

### Scoring

#### Left side score matrices

For each matrix in the left side stack, scoring is carried out almost exactly as described in traditional Smith-Waterman where *A* for all matrices is one candidate fusion read and *B* for each given matrix is the left side reference sequence corresponding to the given index *f* of that matrix. However, calculation of the left side matrices differs slightly from traditional Smith-Waterman in that no score is computed for the final column of each matrix. Instead, as described next, the last base of each left side reference is used to generate a “breakpoint column” corresponding to the fusion breakpoint to which all left-side score matrices are then forced to converge.

### Breakpoint column

The elements of the breakpoint column are calculated by taking the maximum over all possible moves out of the second to the last column of each left side matrix. The center column will be represented by:

$$ {H}_{bkpt}(i) $$

The breakpoint column is populated as follows:

Initialization:

$$ {H}_{bkpt}(0)=0 $$

Scoring:

$$ {H}_{bkpt}(i)=\mathit{\max}\left\{\begin{array}{c}\begin{array}{cc}0& \\ {}{\max}_{1\le \mathrm{f}\le \mathrm{L}}\left\{{H}_{left}\left(i-1,n-1;f\right)+\sigma \left({a}_i,{b}_{n,f}\right)\ \right\}& Match\ or\ Mismatch\end{array}\\ {}\begin{array}{cc}{\max}_{1\le f\le L}\left\{{H}_{left}\left(i,n-1;f\right)+W\right\}& \kern5.5em Deletion\\ {}{H}_{bkpt}\left(i-1\right)+W\kern0.5em & \kern5.75em Insertion\end{array}\end{array}\right\},{\displaystyle \begin{array}{c}\\ {}1\le i\le m\\ {}\end{array}} $$

### Right side score matrices

A score matrix is then generated for each sequence provided as a right side reference. The first column of each right side score matrix is initialized with the values of the breakpoint column, effectively causing each right side matrix to fan out from the breakpoint column. After this initialization step, all of the right side matrices are populated with scores exactly as described in traditional Smith-Waterman scoring where *A* for all matrices is one candidate fusion read and *B* for each given matrix is the right side reference sequence corresponding to the given index *f* of that matrix.

### Best strand

Once all of the score matrices and the breakpoint column are populated, the maximum matrix element over all *H*_*s*_(*i*, *j*; *f*) and *H*_*bkpt*_(*i*) is calculated and stored. FuSpot then generates a full set of fanned score matrices for the read’s reverse complement strand and calculates the maximum matrix element again. The read strand with a higher maximum value has more total matches against the reference sequences and so this strand of the read along with its corresponding set of score matrices passes to the next step to provide the best possible alignment. If the reverse complemented sequence of an original read is selected, that read’s strand is marked as “-” in the final FuSpot plot.

### FuSpot backtrack and alignment

The fanned backtrack mechanism employed by FuSpot is very similar to that of the traditional Smith-Waterman algorithm but is built to handle traversal of the breakpoint column. As with traditional Smith-Waterman backtracking, the process begins at the maximum matrix element, previously calculated while determining the best read strand. Noting the fanned structure of FuSpot alignment, this maximum element also determines the side and index of the matrix from which backtracking commences. If the maximum exists in the breakpoint column backtracking commences from that element of the breakpoint column. The starting position is calculated as follows:

$$ \left({i}_0,{j}_0,{f}_0,S\right)=\left(i,j,f,S\right)\kern0.5em where\kern0.5em {H}_s\left(i,j;f\right)=\max \left\{\begin{array}{c}\max \left\{{H}_{left}\left({i}_x,{j}_{y1};{f}_{z1}\right)\right\}\\ {}\max \left\{{H}_{bkpt}(i)\right\}\\ {}\max \left\{{H}_{right}\left({i}_x,{j}_{y2};{f}_{z2}\right)\right\}\end{array}\right\},\kern0.5em {\displaystyle \begin{array}{c}1\le i\le m,1\le {j}_{y1}\le {n}_1,1\le {j}_{y2}\le {n}_2\ \\ {}1\le {f}_{z1}\le L,1\le {f}_{z2}\le R\ \end{array}} $$

Where:

*f*_*z*1_=*z*_1_th index of the left side matrices.

*f*_*z*2_= *z*_2_th index of the right side matrices.

*L*=number of left side references.

*R*=number of right side references.

(a) If *S* = left:

$$ \left({i}_{q+1},{j}_{q+1},f\right)=\left(i,j,f\right)\  where\ {H}_{left}\left(i,j;f\right)=\max \left\{\begin{array}{cc}{H}_{left}\left({i}_q-1,{j}_q-1;f\right)+\sigma \left({a}_i,{b}_{n,f}\right)\ & Diagonal\\ {}{H}_{left}\left({i}_q,{j}_q-1;f\right)+W& Left\\ {}{H}_{left}\left({i}_q-1,{j}_q;f\right)+W& Above\end{array}\kern0.75em \begin{array}{c}{A}_{out}+={a}_{i_q},{B}_{out}+={b}_{f,{j}_q}\ \\ {}{A}_{out}+={}^{"}-{}^{"},{B}_{out}+={b}_{f,{j}_q}\\ {}{A}_{out}+={a}_{i_q},{B}_{out}+={}^{"}-{}^{"}\kern0.5em \end{array}\right\}, until\kern0.5em {H}_{left}\left(i,j;f\right)=0 $$

(b) If *S* = bkpt; f, j = 0 (starts directly on breakpoint):

$$ \left({i}_{q+1},{j}_{q+1},f,S\right)=\left(i,j,f,S\right)\  where\ {H}_S\left(i,j;f\right)=\max \left\{\begin{array}{cc}{\max}_{1\le {f}_{z1}\le L}\left\{{H}_{left}\left({i}_q-1,{n}_1-1;{f}_{z1}\right)+\sigma \left({a}_i,{b}_{n,f}\right)\right\}& Diagonal\\ {}{\max}_{1\le {f}_{z1}\le L}\left\{{H}_{left}\left({i}_q,{n}_1-1;{f}_{z1}\right)+W\right\}& Left\\ {}{H}_{bkpt}\left({i}_q-1\right)+W& Above\end{array}\kern0.75em \begin{array}{c}{A}_{out}+={a}_{i_q},{B}_{out}+={b}_{f_{z1},{n}_1}\ \\ {}{A}_{out}+={}^{"}-{}^{"},{B}_{out}+={b}_{f_{z1},{n}_1}\\ {}{A}_{out}+={a}_{i_q},{B}_{out}+={}^{"}-{}^{"}\kern0.5em \end{array}\right\}, until\kern0.5em {H}_S\left(i,j;f\right)=0\  or\ S= left $$

If *S* = left and *H*_*S*_(*i*, *j*; *f*) ≠ 0: continue on using step **a**.

(c) If *S* = right (starting in the right-side score matrices):

$$ \left({i}_{q+1},{j}_{q+1},f\right)=\left(i,j,f\right)\  where\ {H}_{right}\left(i,j;f\right)=\max \left\{\begin{array}{cc}{H}_{right}\left({i}_q-1,{j}_q-1;f\right)+\sigma \left({a}_i,{b}_{f,j}\right)& Diagonal\\ {}{H}_{right}\left({i}_q,{j}_q-1;f\right)+W& Left\\ {}{H}_{right}\left({i}_q-1,{j}_q;f\right)+W& Above\end{array}\kern0.75em \begin{array}{c}{A}_{out}+={a}_{i_q},{B}_{out}+={b}_{f,{j}_q}\ \\ {}{A}_{out}+={}^{"}-{}^{"},{B}_{out}+={b}_{f,{j}_q}\\ {}{A}_{out}+={a}_{i_q},{B}_{out}+={}^{"}-{}^{"}\kern0.5em \end{array}\right\}, until\kern0.5em {H}_{right}\left(i,j;f\right)=0\  or\ j=0 $$

If *j* = 0 and *H*_*right*_(*i*, *j*; *f*) ≠ 0: continue on using step **b**, setting (*i*_*q* + 1_, *s*) = (*i*_*q* + 1_, *bkpt*).

**Fig. 3:**
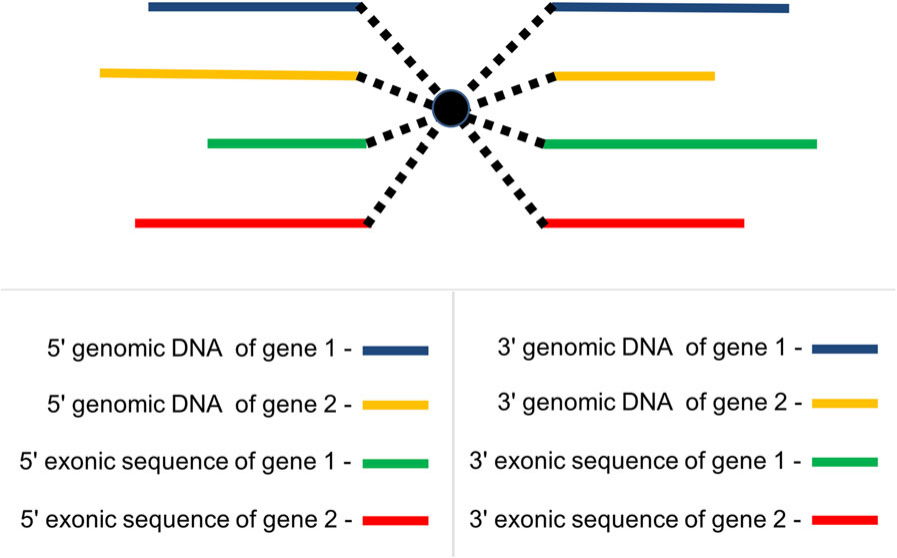
FuSpot Fusion Alignment Algorithm Visualized. A conceptual representation of the FuSpot alignment algorithm. Each colored line represents an overhead view of a 2D Smith Waterman score matrix representative of a given read and one of eight reference sequences (4 on the 5′ end of the breakpoint and 4 on the 3′ end.) The central black circle marks the fusion breakpoint. Since fusion analysis is typically conducted with RNA data, the ability to align against many references simultaneously is crucial. Here the chosen references exemplify how FuSpot can be used to align a set of reads, each of which may be fusion or non-fusion as well as genomic or exonic. During FuSpot realignment, should backtracking commence from a matrix on the 3′ side of the breakpoint, tracing could follow through to the breakpoint. In such a case, FuSpot’s realignment algorithm would search the rightmost column of each of the four matrices on the 5′ end of the breakpoint for the appropriate next step to trace. Once determined, it will follow the dotted line to that matrix, then trace through it for the remainder of the backtrack. Subsequently, the traced 5′ and 3′ matrices and their associated references will be assigned a color during FuSpot’s visualization step and the aligned read will take on the appropriate color for the reference to which it aligned on either side of the breakpoint

Once step a, b, or c encounters a matrix element of 0, backtracking ceases and the finalized alignments are saved. This special fanned technique allows a user to query the alignment of a given read against numerous reference sequences simultaneously. This is particularly important when working with RNA data. In Fig. 3 we conceptualize this alignment method and show an example of how FuSpot can be used to locally align an entire set of reads, each of which may be a fusion or non-fusion read as well as a genomic or exonic read.

### Alignment score

Once alignment is finished, each read is assigned an alignment score to track the quality of the reported alignment. If a read aligns perfectly somewhere along the references, it receives a score of 100. However, FuSpot’s alignment algorithm allows for any number of insertions, deletions and mismatches, each of which can contribute negatively to a read’s final alignment score. This metric is valuable as a filtering parameter on the web interface and will be discussed in more detail in the next section.

Alignment score is calculated as follows:

$$ \alpha =\frac{H_s\left({i}_0,{j}_0;{f}_0\right)}{m}\ast 100 $$

Where:

- *H*_*s*_(*i*_0_, *j*_0_, *f*_0_)=value of the maximum matrix element where backtracking commenced
- *m*=length of the read

### FuSpot web interface

Once every read is assigned an alignment and a score, all reads are then traced and colored on a user-friendly web platform. References on each side of the breakpoint are assigned a unique color, and the reads that align to that reference will take on the same color. As such, fusion reads are readily identifiable since any read that aligns across the breakpoint will take on two distinct colors. By clicking a read, the user will see all information associated with it, including the read name, the input strand orientation, and its alignment score.

FuSpot allows two options for filtering read alignments. First, the user may dynamically set a minimum alignment score to examine the quality of the alignments of their reads. This becomes useful in ascertaining whether the supporting reads reported by a fusion tool truly align well to the fusion breakpoint in question. This is also valuable for quickly filtering out input reads that did not align well to any of the reference sequences. Second, the user has the option to view only spanning reads. In most cases, a minimum of two spanning reads is required by fusion detector tools to report a fusion. This is because without any spanning reads, it is impossible to know the exact position of the breakpoint. Using this option, the user can quickly discern the number of these important reads in their data.

### Additional features

#### Paired-end

FuSpot is able to align and display paired-end reads. During alignment, read-pair information is ignored and all reads are aligned independently. However, the read-pair information is utilized in the web interface to plot mates along the same line. During filtering, the alignment score of each read in a pair must be greater than the minimum alignment score for the pair to be displayed. If the user selects the spanning filter option, read pairs that have at least one read spanning the fusion breakpoint will be displayed.

### Semi-global alignment

FuSpot may also be run in semi-global alignment mode. Users may prefer this option since it guarantees that the entire read will be traced during the backtrack step and that every base will be used in the reported alignment. This is achieved by forcing the backtracking mechanism to begin on the bottom row of a matrix, and only cease execution when the top row of a matrix is encountered. The process is as follows:

### Scoring

Scoring for left side matrices, the breakpoint column and right side score matrices is calculated using the same method as described in *FuSpot Fanned Alignment: Left Side Score Matrices, FuSpot Fanned Alignment: Breakpoint Column,* and *FuSpot Fanned Alignment: Right Side Score Matrices* respectively, with the following two exceptions: matrix elements are permitted to become negative (i.e. the *floor* value is omitted from input to the max function). In addition, the left side score matrices are initialized as follows:

$$ {H}_{left}\left(i,0,f\right)=-i,\kern0.75em 0\le i\le m $$

$$ {H}_{left}\left(0,j,f\right)=0,\kern0.75em 0\le j\le n $$

$$ 0\le f\le L $$

### Starting matrix element

The matrix element on which to begin the backtrack scheme is calculated as follows:

$$ \left({i}_0,{j}_0,{f}_0,s\right)=\left(i,j,f,s\right)\kern0.5em where\kern0.5em {H}_s\left(i,j,f\right)=\max \left\{\begin{array}{c}\max \left\{{H}_{left}\left(m,{j}_{y1},{f}_{z1}\right)\right\}\\ {}{H}_{bkpt}\left(m,0,0\right)\\ {}\max \left\{{H}_{right}\left(m,{j}_{y2},{f}_{z2}\right)\right\}\end{array}\right\},\kern0.5em {\displaystyle \begin{array}{c}1\le {j}_{y1}\le {n}_1,1\le {j}_{y2}\le {n}_2\ \\ {}0\le {f}_{z1}\le L,0\le {f}_{z2}\le R\ \end{array}} $$

### Backtrack termination

Backtracking only terminates when the top row of a matrix is reached:

$$ {i}_q=0 $$

### Alignment score

The value of the starting matrix element of the semi-global backtrack mechanism is used when calculating the alignment score in semi-global mode:

$$ {H}_s\left({i}_0,{j}_0;{f}_0\right) $$

## Results and discussion

Since, to the best of our knowledge, no other tool exists to visualize fusion read alignments, we evaluated the performance and utility of FuSpot by examining data supporting gene fusions whose validity was known a priori. Given the frequency at which new fusion tools are being developed, FuSpot is designed to analyze putative fusion candidates independent of the detector tool that reported them. To demonstrate this versatility, herein we present cases in which we run four different fusion detectors on two publicly available data sets, and use FuSpot to validate the reported true and false positives.

Many factors influence the performance of any given fusion detection algorithm; the most important of these are the characteristics of the input data. These include read length, strandedness, insert length, sequencing coverage, whether the data is paired-end or single-end, and whether the data is deoxyribonucleic acid (DNA) or RNA. When evaluating the performance of a given tool, the most important considerations are its run time, memory footprint, sensitivity and specificity. To present a diverse set of cases in which a researcher might carry out a fusion study, we selected four tools (FusionCatcher [18], FusionMap [19], EricScript [20], and Bellerophontes [21]) that were reported to vary significantly in these categories, and that recent reviews [8, 9] agreed were the most effective over diverse data sets. Per Liu et al. and Kumar et al. [8, 9], FusionCatcher has good precision and sensitivity but a relatively higher computation cost and run time whereas FusionMap has slightly lower precision and sensitivity but a very low run time. These tools were also selected because they included supporting reads for candidate fusion junctions in their output files. For simplicity we ran only these two tools on our positive data set. For the negative dataset, we ran these two tools as well as EricScript and Bellerophontes in order to demonstrate the diverse false positive populations that each tool may report -- and which FuSpot can effectively analyze. EricScript has a runtime that rivals FusionMap, a small memory footprint, and relatively high predictive power, whereas Bellerophontes has similar predictive power with higher computation cost and runtime [8, 9].

First, we ran FusionCatcher and FusionMap on a publicly available paired-end RNA Sequencing (RNA-Seq) data set derived from the BT474 breast cancer cell line, used in Edgren et al. [22] and available on the National Center for Biotechnology Information (NCBI) Sequence Read Archive (SRA) [SRA:SRP003186]. This data set contains several polymerase chain reaction (PCR) validated gene fusions and has been used as a test set in other fusion detector publications [18, 20, 21, 23, 24]. To search for false positives, FusionCatcher, FusionMap, EricScript, and Bellerophontes were also run on a publicly available synthetic paired-end RNA-Seq data set generated using the Benchmarker for Evaluating the Effectiveness of RNA-Seq Software (BEERS) [25]. This dataset is claimed to be free of fusion events and used in the publication of the JAFFA fusion detector tool [26] and is available on the JAFFA website [27]. Since this data set is built to contain no true fusions, any fusion junction events reported by the selected fusion tools should be regarded as false positives.

To provide more information adjacent to the fusion junctions reported by the four tools, we also aligned both data sets using the STAR RNA aligner [16] in chimeric mode, then extracted with FuSpot reads which STAR reported to align in the vicinity of each breakpoint. In chimeric mode, STAR provides both chimeric and normal RNA alignments thereby allowing us to gather reads from both fusion and non-fusion gene transcripts. By visualizing both types of reads in FuSpot, researchers can gain a more incisive view of the fusion junctions, which is not possible by viewing just fusion-supporting reads alone.

For the following fusion junctions, 200 bp genomic and exonic sequences on either side of the breakpoints were used as references for FuSpot alignment and visualization.

### True positive: ACACA-STAC2

Edgren et al. [22] reported 10 PCR validated true positive fusions in the BT474 data set. Of these, the ACACA-STAC2 fusion gene had the highest read coverage and was therefore the best candidate to comprehensively illustrate the functionality of FuSpot. We ran FusionCatcher and FusionMap using their default parameters on the Edgren et al. [22] data set and both reported ACACA-STAC2 as a fusion candidate. We also ran STAR with chimeric options enabled to identify a separate set of fusion reads local to the breakpoint.

### FuSpot: Supporting reads by FusionMap

**Fig. 4:**
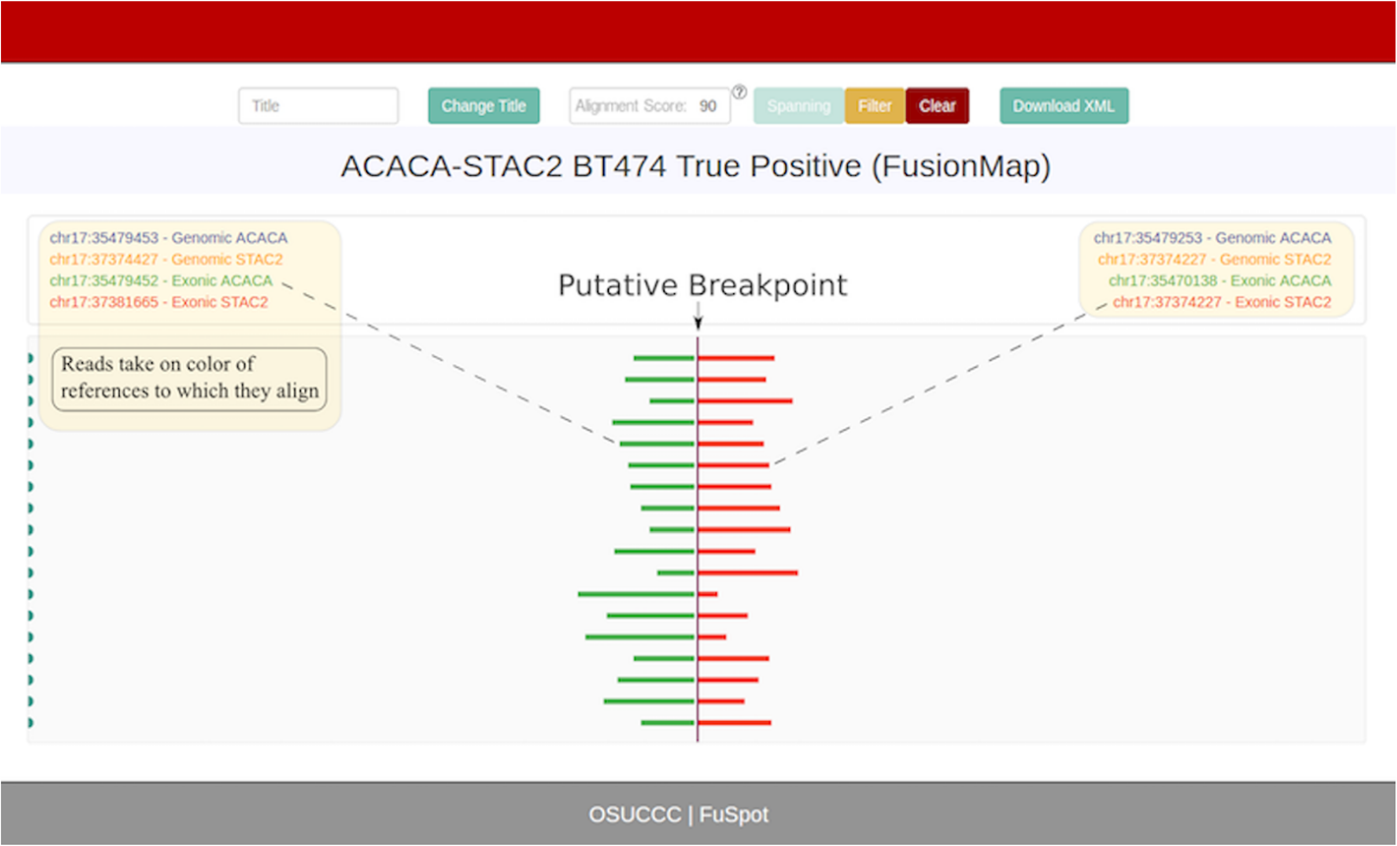
FuSpot Visualization of ACACA-STAC2 True Fusion Gene Using FusionMap Supporting Reads. FuSpot’s visualization of the lab-validated ACACA-STAC2 fusion gene from Edgren et al. [22] The breakpoint for the fusion is marked by the dotted line in the center of the plot. Each colored horizontal line in the plot corresponds to one read local to the fusion breakpoint that was realigned by FuSpot. On either side of the breakpoint, references used for realignment are assigned a unique color in the legend atop the plot and the reads that align to each reference will take on the same color. Each read seen here was reported by the FusionMap fusion detector tool when run on the data set from Edgren et al. [22] As supporting read evidence, FusionMap provides exclusively single-end reads which align over the breakpoint. The even distribution of these reads about the breakpoint support the existence of the candidate fusion, but the omission of the mates leaves the picture incomplete; specifically, it excludes any reads which, along with their paired mates, may align on either side of the breakpoint

**Fig. 5:**
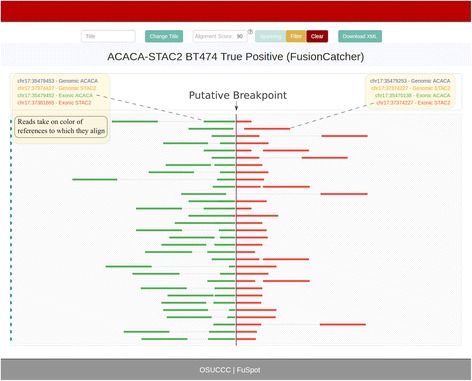
FuSpot Visualization of ACACA-STAC2 True Fusion Gene Using FusionCatcher Supporting Reads. An alternative visualization produced by FuSpot of the lab-validated ACACA-STAC2 fusion gene from Edgren et al. [22] Each read seen here was reported by the FusionCatcher fusion detector tool when run on the data set from Edgren et al. [22] Unlike FusionMap, FusionCatcher provides in its output supporting reads and available mates which span or flank the breakpoint. Here, several reads span the breakpoint, some with a spanning first mate and some with a spanning second. Anchor lengths over the breakpoint vary from a few base pairs to about half the length of the read. Two reads flank the breakpoint. This distribution provides clear and present evidence that the ACACA-STAC2 fusion is a true positive

**Fig. 6:**
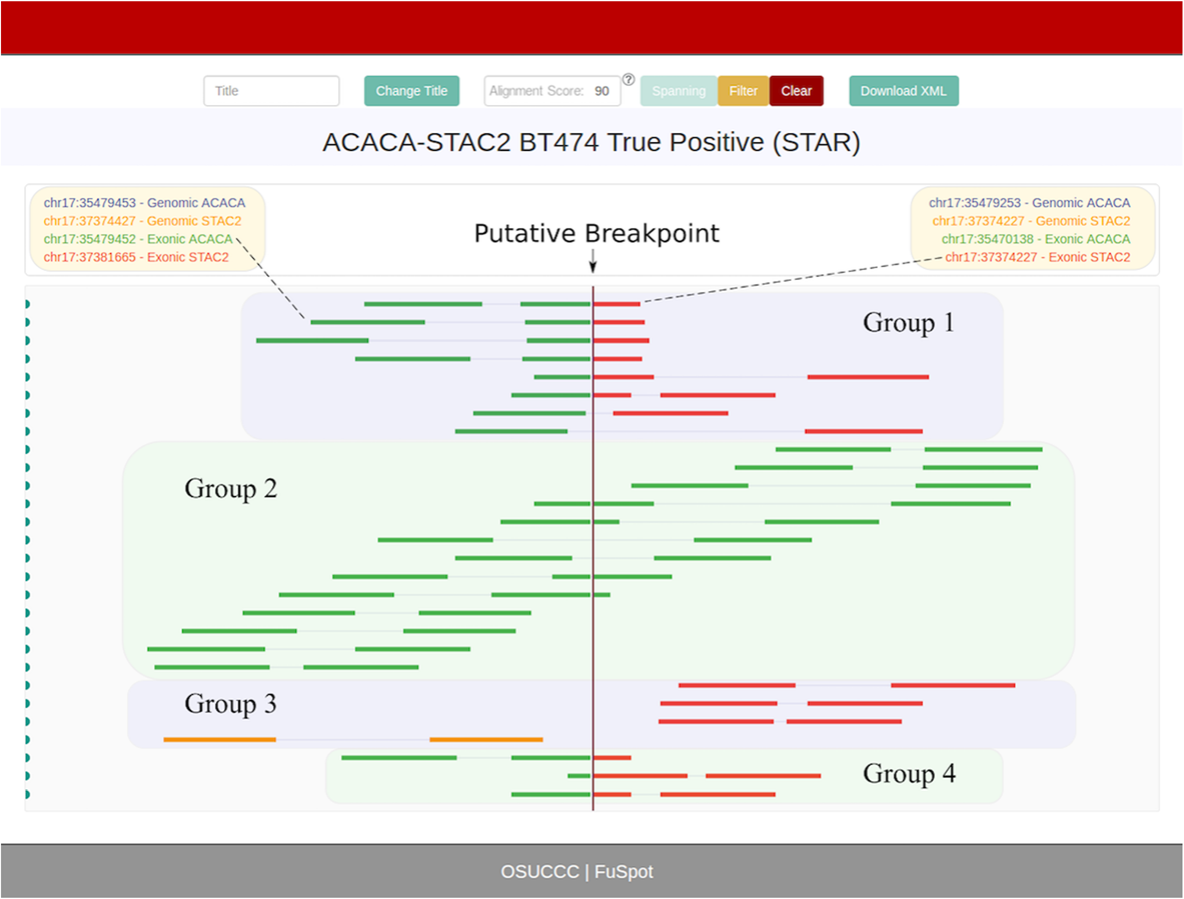
FuSpot Visualization of ACACA-STAC2 True Fusion Gene Using Extracted STAR Supporting Reads. FuSpot’s final visualization of the lab-validated ACACA-STAC2 fusion gene from Edgren et al. [22] Each read seen here was extracted from either the normal or chimeric output file produced by aligning the data set from Edgren et al. [22] with STAR. All reads were extracted using the FuSpot extraction tool available on the FuSpot website. In the plot, these reads make up 4 distinct groups. Group 1 is made up of chimeric reads which represent a clear expression of the putative fusion gene. Groups 2 and 3 contain reads extracted from STAR’s normal alignment file which support the normal ACACA and normal STAC2 transcripts respectively. Group 4 is made up of reads that were extracted from the normal alignment file, but that are truly chimeric reads. These four groups exemplify the utility of FuSpot in building a comprehensive visual representation of a putative fusion

FusionMap includes in its output a list of supporting reads that align over the implicated fusion breakpoint. However, these supporting reads are exclusively single-ended and therefore lack valuable information about the fusion as we will show below. Figure 4 depicts the FuSpot visualization of reads reported by FusionMap supporting the ACACA-STAC2 fusion junction. All 18 of these supporting reads achieved at least a 95/100 FuSpot alignment score. The reads span the breakpoint evenly providing compelling evidence that this is likely a true fusion candidate. However, as read mates are not part of the FusionMap output, reads flanking the breakpoint are not present to build a comprehensive view to fully support the assertion. In Fig. 5 and Fig. 6, we illustrate the power of including flanking reads in FuSpot to further augment the evidence of the presence of ACACA-STAC2 fusion in the data.

### FuSpot: Supporting reads by FusionCatcher

The other fusion tool we evaluated on the positive data set was FusionCatcher. As supporting evidence, this tool provides reads with mates spanning or flanking the fusion point. Figure 5 depicts FuSpot’s output showing the FusionCatcher reads supporting the ACACA-STAC2 fusion junction. Of the 87 read pairs provided by FusionCatcher as supporting reads, 74 were aligned by FuSpot with a 95/100 alignment score or higher. These include 30 read pairs which have the first mate align in ACACA and the second mate aligning over the gap into STAC2. Conversely, 11 read pairs align with the first mate aligning over the gap and the second mate aligning fully in STAC2. Further, the anchor lengths of spanning reads vary from a few base pairs up to 25 bp on each side of the gap. Finally, 33 reads flank the breakpoint such that each mate lies in a unique gene without aligning over the breakpoint. In all, the visual display of candidate RNA-seq reads to the ACACA-STAC2 chimeric gene illustrate all the characteristics expected of a true fusion gene. This clearly highlights the value of FuSpot as an accompanying tool to fusion detection algorithms.

### FuSpot: Supporting reads by STAR

Figure 6 shows FuSpot’s output depicting the STAR alignment reads adjacent to the ACACA-STAC2 fusion junction. The reads were extracted from both the normal and chimeric alignment files and make up 4 distinct groups. Group 1 is made up of chimeric reads which represent a clear expression of the putative fusion gene, similar to the reads in Fig. 5. Groups 2 and 3 contain reads extracted from STAR’s normal alignment file. As seen in the figure, these reads are not involved in the fusion transcripts – rather they support the normal ACACA and normal STAC2 transcripts respectively. Such reads are expected in fusion sequencing data since a fusion is usually present in only one copy of a given chromosome. Group 4 is made up of reads that were extracted from the normal alignment file, but that are truly chimeric reads. These reads were placed in the normal alignment file since STAR successfully aligned them with many bases in the first or second mate soft clipped. When these soft clipped reads were aligned by FuSpot, they were revealed as chimeric reads supporting the putative fusion. These four groups exemplify the utility of FuSpot in building a comprehensive visual representation of a putative fusion using all the data available to the researcher.

### False positives

Next, we ran FusionCatcher, FusionMap, EricScript, and Bellerophontes with default options on the BEERS [25] data set in order to discover false positive fusion points. The number of false positives reported by each tool can be seen below in Table 1.

**Table 1:** False Positives Reported by the Fusion Detectors

Tool	False Positives	Large Scale FP
FusionCatcher	142	1
FusionMap	5	3
EricScript	298*	137
Bellerophontes	5060	4984

We ran FusionCatcher, FusionMap, EricScript, and Bellerophontes on the synthetically generated BEERS [25] data set known not to contain any true gene fusions. The False Positives column indicates the total number of fusions reported by each tool. The Large Scale FP column depicts the number of reported fusions that involved gene partners at least 100 kilobase pairs apart or on different chromosomes (all others were considered read-throughs rather than potential genomic relocations.)

*The total number of false positives for EricScript was counted using the reported list of fusions containing EricScore > 0.5 [20]

FusionCatcher detected 142 fusion events, 141 of which were labeled as having a gap of less than 100 kilobase pairs (kb). The relatively small distance between the breakpoints suggested that these events were transcriptional read-throughs rather than potential genomic relocations. Thus these breakpoints were eliminated from the final analysis with FuSpot. Alternatively, FusionMap detected 5 fusion events, 2 of which were on the same chromosome spanning a gap less than 100 kb. One of the three remaining fusions suggested the presence of a breakpoint between Chr3 and Chr1 and provided four spanning supporting reads as evidence. The fusion gene connected THRB and AZIN2 (formerly known as ADC). We chose to first investigate this gene with FuSpot due to the significant level of supporting evidence and the scale of the relocation event.

### **THRB-AZIN2**: Supporting reads by FusionMap

**Fig. 7:**
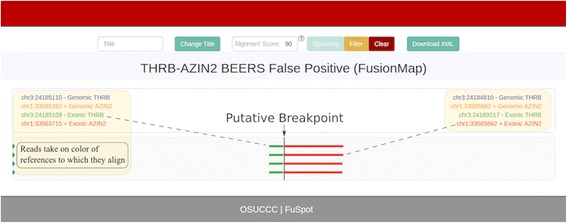
FuSpot Visualization of THRB-AZIN2 False Positive Fusion Junction Using FusionMap Supporting Reads. FuSpot’s visualization of the putative THRB-AZIN2 fusion gene claimed by FusionMap when given the BEERS data set [25]. Each read seen here was reported by the FusionMap fusion detector tool when run on the above-mentioned data set. To begin, FusionMap identified 4 supporting reads. Inspection in FuSpot revealed that the second and fourth reads were reverse complemented copies of the first and third reads respectively, diminishing the amount of reliable supporting evidence. This evidence alone is insufficient to draw a meaningful conclusion about the validity of the fusion

Figure 7 shows the FuSpot alignment of the four putative supporting reads provided by FusionMap to the THRB-AZIN2 fusion gene. Inspection in FuSpot revealed that the second and fourth reads were reverse complemented copies of the first and third reads respectively, further diminishing the amount of reliable supporting evidence. The lack of a distribution among the supporting reads and consistent short anchor length suggest that this candidate breakpoint may be a false positive, matching the hallmarks of false positives reported by Edgren et al. [22]

### **THRB-AZIN2**: Supporting reads by STAR chimeric

**Fig. 8:**
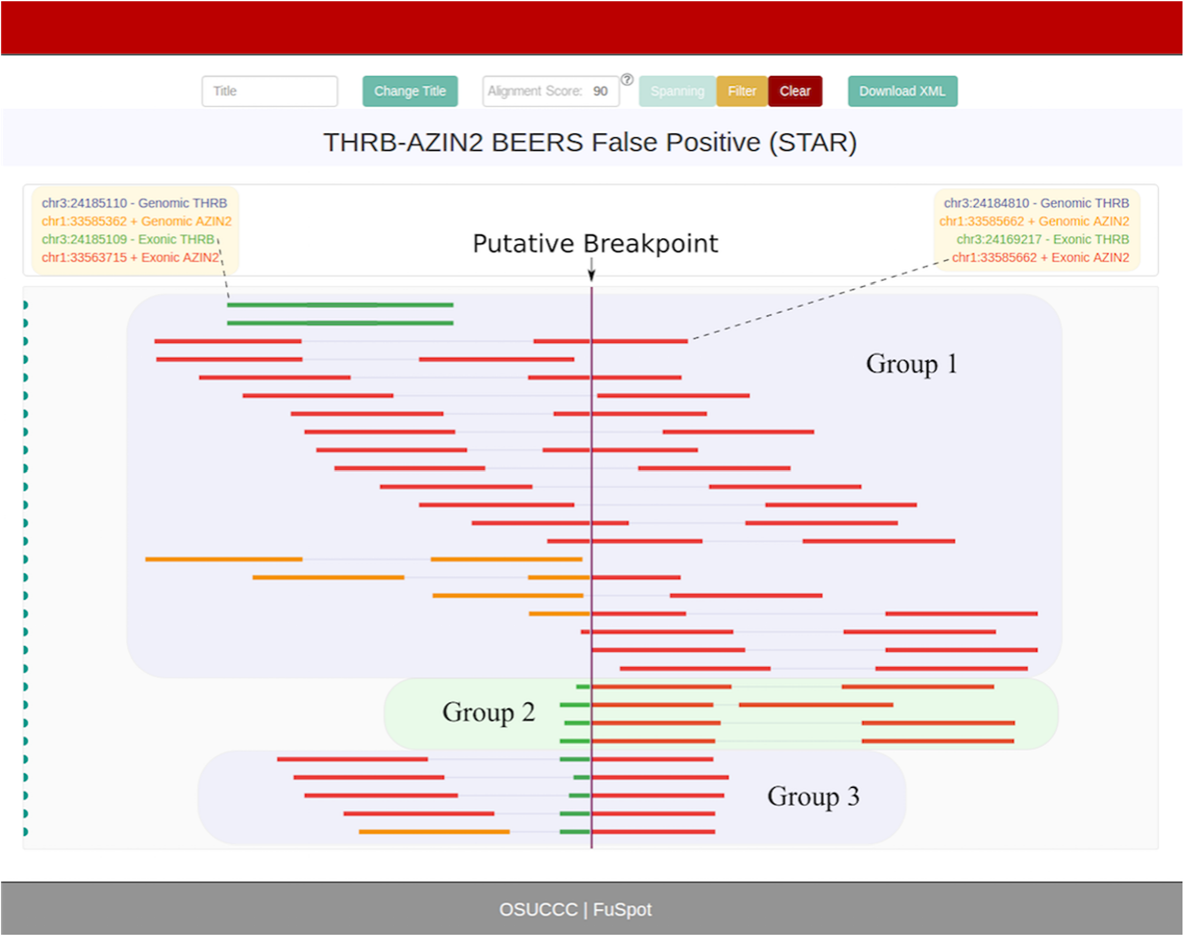
FuSpot Visualization of THRB-AZIN2 False Positive Fusion Junction Using Extracted STAR Supporting Reads. FuSpot’s alternative visualization of the putative THRB-AZIN2 fusion gene claimed by FusionMap when given the BEERS data set [25]. Each read seen here was extracted from either the normal or chimeric output file produced by aligning the BEERS [25] data set with STAR. All reads were extracted using the FuSpot extraction tool available on the FuSpot website. STAR’s chimeric file yielded no reads that aligned near the breakpoint with at least a 95/100 score percentage. However, STAR’s normal alignment file contained many non-fusion reads (Group 1) which together form an even distribution across the AZIN2 gene. Four reads (Group 2) align such that the first mate spans the fusion breakpoint with a small anchor on the 5′ end and the second mate aligns entirely in the 3′ end of the AZIN2 reference. Below these are five reads (Group 3) that align such that the first mate aligns fully in the 5′ end of the AZIN2 reference and the second mate aligns such that its first few bases align to the 5′ end of the THRB reference and the remaining bases align to the 3′ end of the AZIN2 reference. These “nonsense alignments” in Group 3 suggest that there is homology between the sequence at the terminus of the 5′ end of the THRB reference and the sequence at the terminus of the 5′ end of the AZIN2 reference. This homology is confirmed in Fig. 9 by aligning Groups 2 and 3 to only AZIN2 references

**Fig. 9:**
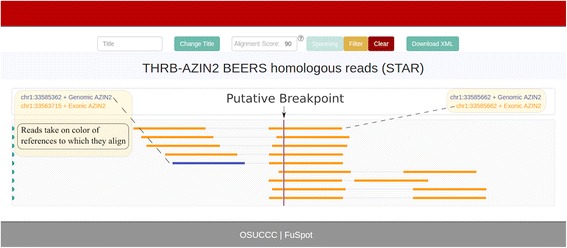
FuSpot Alignment of THRB-AZIN2 Supporting Reads to only AZIN2 References: FuSpot’s visualization of reads from Groups 2 and 3 of Fig. 8 realigned to only AZIN2 references. In Fig. 8, all reads of Groups 2 and 3 aligned to their respective references with at least at 95/100 alignment score. Similarly, in this plot, all reads align with at least a score of 94/100. This shows the homology between the AZIN2 and THRB reference sequences and provides evidence as to why this false fusion candidate was reported by FusionMap

Figure 8 shows the FuSpot alignment of reads reported by STAR to align near the THRB-AZIN2 fusion point. Extraction and examination of fusion reads from STAR’s chimeric file yielded no reads that aligned with at least a 95/100 score percentage. However, STAR’s normal alignment file contained many reads, which we will call Group 1, that were local to the AZIN2 breakpoint coordinate as depicted by FuSpot in Fig. 8. Most of the reads were non-fusion reads with an even distribution across the AZIN2 gene. Four reads near the bottom of the plot (which we will call Group 2) align such that the first mate spans the fusion breakpoint with a small anchor on the 5′ end and the second mate aligns entirely in the 3′ end of the AZIN2 reference. Below these are five reads, which we will call Group 3, that align such that the first mate aligns fully in the 5′ end of the AZIN2 reference and the second mate aligns such that its first few bases align to the 5′ end of the THRB reference and the remaining bases align to the 3′ end of the AZIN2 reference, defying genome orientations. These “nonsense alignments” reveal why FusionMap reported this breakpoint. The Group 3 reads suggest that there is homology between the sequence at the terminus of the 5′ end of the THRB reference and the sequence at the terminus of the 5′ end of the AZIN2 reference. To confirm this homology, we aligned the reads from Groups 2 and 3 with FuSpot using only the AZIN2 sequences as references. All 9 reads aligned with a 94/100 or greater score (see Fig. 9). This similarity between the references likely caused FusionMap to misalign the four reads from Group 1 over the breakpoint rather than over the true AZIN2 gene, resulting in false supportive reads. This illustrates the functionality of FuSpot to inspect putative fusion reads by inspecting three different lines of evidence (Groups 1, 2, and 3 reads) thereby allowing us to mark this candidate breakpoint with confidence as a false positive fusion candidate.

### DNAJC21-CNGA1: EricScript false positive

**Fig. 10:**
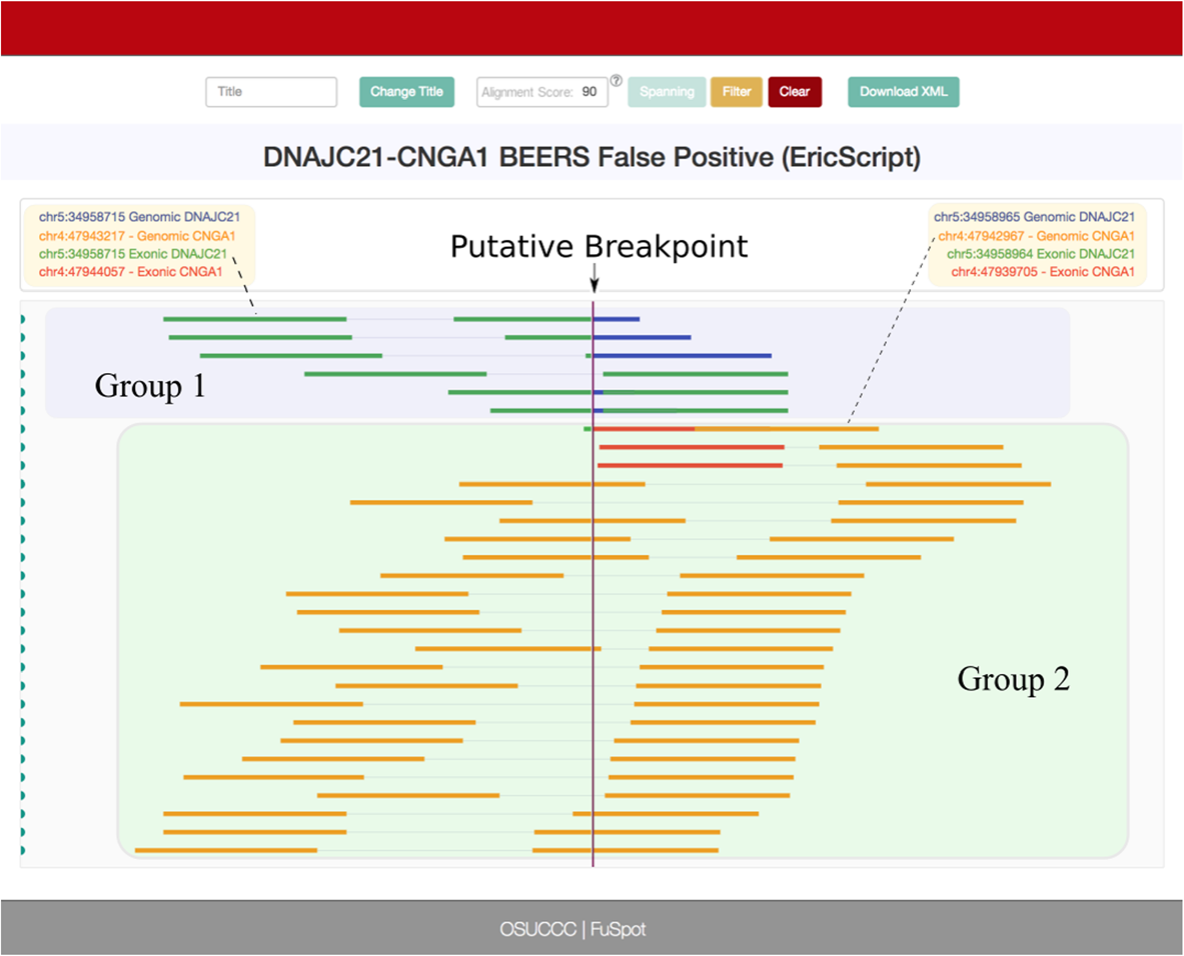
FuSpot Visualization of DNAJC21-CNGA1 False Positive Fusion Junction Reported by EricScript. FuSpot’s visualization of the putative DNAJC21-CNGA1 fusion gene claimed by EricScript when given the BEERS data set [25]. Each read seen here was extracted from either the normal or chimeric output file produced by aligning the BEERS [25] data set with STAR. All reads were extracted using the FuSpot extraction tool available on the FuSpot website. The reads in Group 1 all align normally across the DNAJC21 partners and the majority of reads in Group 2 align in the congruent manner across the CNGA1 partners. However, through FuSpot’s realignment, the first read of Group 2 reveals that there is sequence homology along the last few base pairs of the 5′ references; this is likely what caused EricScript to falsely report the fusion point

EricScript generally requires low run time and computation resources and is therefore a promising tool for general purpose fusion detection. However, it returned a larger number of false positives than either FusionCatcher or FusionMap when presented with the synthetic BEERS [25] data set, underscoring the value and need for FuSpot in evaluating its fusion candidates. Figure 10 shows the FuSpot alignment of reads reported by STAR to align near the EricScript’s falsely reported DNAJC21-CNGA1 fusion point. The reads in Group 1 all align normally across the DNAJC21 partners and the majority of reads in Group 2 align in a congruent manner across the CNGA1 partners. However, through FuSpot’s realignment of the putative supporting reads, the alignment of the first Group 2 read reveals that there is sequence homology among the last few base pairs of the 5′ references; this is likely what caused EricScript to falsely report the fusion point. The plot visually confirms that there is insufficient evidence in the underlying data to support the presence of this fusion.

### VDAC1-VDACP2: Bellerophontes false positive

**Fig. 11:**
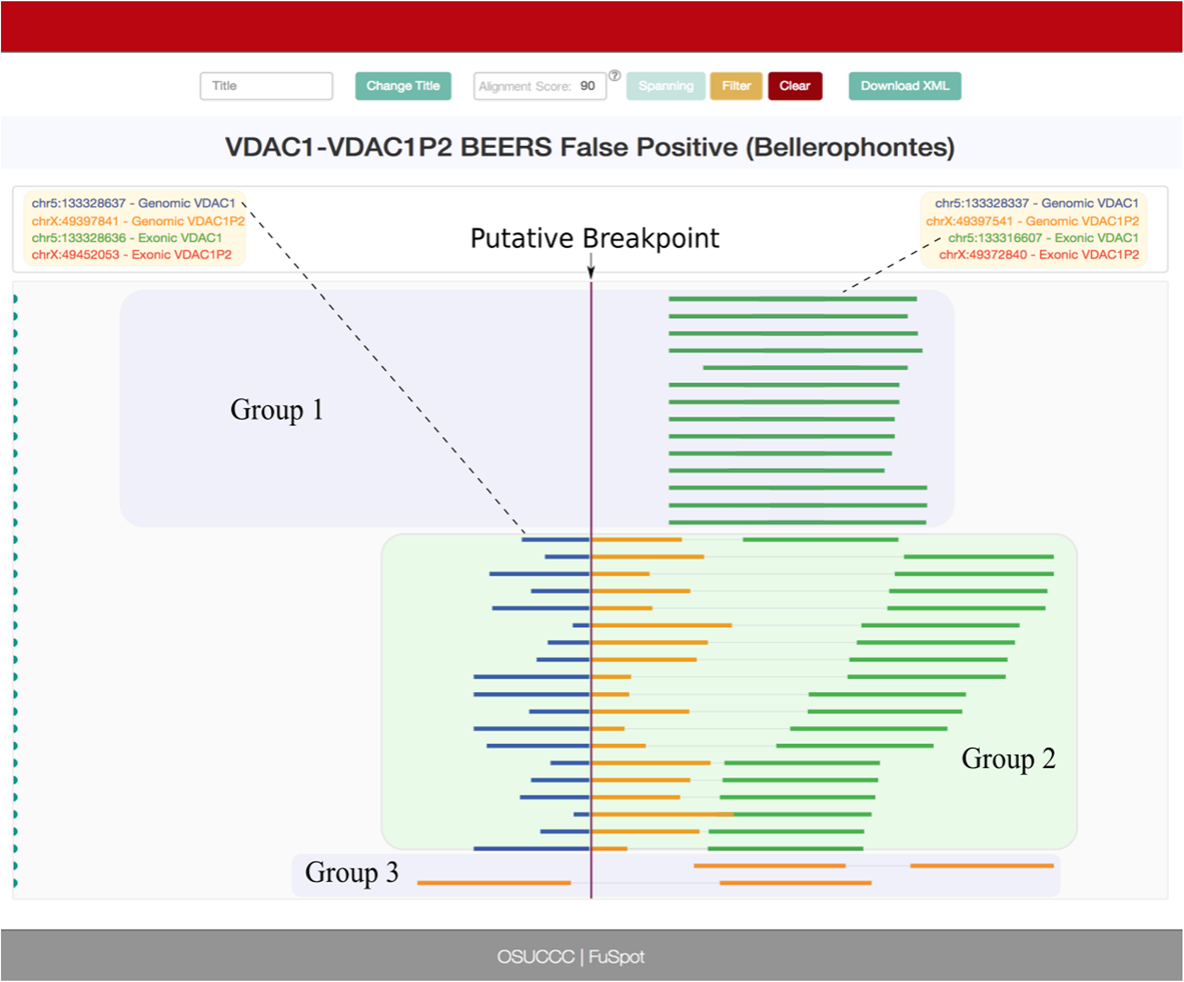
FuSpot Visualization of VDAC1-VDAC1P2 False Positive Fusion Junction Reported by Bellerophontes. FuSpot’s visualization of the putative VDAC1-VDAC1P2 fusion gene claimed by Bellerophontes when given the BEERS data set [25]. Each read seen here was extracted from either the normal or chimeric output file produced by aligning the BEERS [25] data set with STAR. The reads in Group 1 and 3 align as non-fusion reads to their respective gene partners. However, FuSpot’s realignment and visualization exposes the reads in Group 2 which all lack biological significance. Similar to the THRB-AZIN2 fusion point in Fig. 8, these alignments show that the VDAC1 and VDAC1P2 sequences on the 3′ side of the breakpoint are highly similar and is likely what caused the tool to report the fusion candidate. We can visually reject this candidate from downstream analysis

Bellerophontes, like EricScript, was ranked highly in recent reviews for its overall performance in fusion detection across the four main performance categories. However, the performance of Bellerophontes on the BEERS [25] synthetic dataset reveals its low specificity, resulting in the greatest number of false positives of all the tested tools, further underscoring the need for FuSpot to visualize outputs from current fusion detectors. Figure 11 shows the FuSpot alignment of reads reported by STAR to align near the falsely reported VDAC1-VDAC1P2 fusion point. The reads in Group 1 and 3 align as non-fusion reads to their respective gene partners. However, FuSpot’s realignment and visualization exposes the reads in Group 2 which all lack biological significance. Similar to the THRB-AZIN2 fusion point above, these alignments show that the VDAC1 and VDAC1P2 sequences on the 3′ side of the breakpoint are highly similar and is likely what caused the tool to report the fusion candidate. Once again, FuSpot enables us to visually confirm that this fusion point can be eliminated from downstream analysis.

### Impact

Despite much advancement in fusion detection algorithms, no tool has emerged as the gold standard for this area of research. Currently, each fusion detector tool offers unique features that researchers may want to utilize for their specific study design and focus. Some studies may demand high sensitivity, others absolute precision and most would appreciate computation speed. Most importantly, researchers need a way to remove false positives and to prioritize potential true positives so as to identify the most promising set of true positive fusion candidates for laboratory-based gene fusions validations. A recently published tool, chimeraviz [28], can help with this prioritization. This R package visualizes the metrics provided by any of nine modern fusion detectors to help users evaluate fusion candidates. This can be useful to prioritize which candidates to analyze on a detector by detector basis. However, since chimeraviz uses as its evidence exactly the data reported by a given detector, its visualizations will be subject to the same biases or underlying errors that the tools themselves report. FuSpot offers greater evaluative power than chimeraviz because it realigns all reads adjacent to a given fusion candidate’s breakpoint, exposing faults (such as sequence homology) that often cause detectors to report false positives.

FuSpot purposefully does not attempt to combine the evidence for or against a fusion candidate into a single one-dimensional score or *p*-value, which then could be subjected to a cutoff that separates true from false positives. FuSpot’s philosophy is rather that evidence for a fusion is intrinsically multidimensional and that a holistic review of intuitively presented evidence by a human expert is superior to any given scoring system as the final step in the prioritization of fusion candidates for experimental validation. As explained in the examples above, evidence for true positives includes multiple reads that switch reference at the breakpoint with switching-points well distributed over the entire lengths of the reads and with consistent partners in the read pair. On the contrary, when one of the two gene partners only occupies a small and consistent fraction of the switching reads, this is likely an indication of a false positive caused by a sequence homology; reads that switch from one gene to the other in opposite directions or where the partner in the read pair is placed inconsistently, are even stronger indicators of false positives.

Ultimately, FuSpot gives researchers the power to visualize and qualitatively determine the validity of reported fusions regardless of the detection tool they use. If a detection tool provides supporting reads as output, users can validate and visualize the reads to gain a much deeper understanding of why the detector reported the fusion point than can be surmised from a tools’ confidence metrics alone. Importantly, if a detection tool does not provide supporting reads, FuSpot will be able to extract the appropriate information from the output of a chimeric aligner such as STAR to build highly detailed visualizations around the breakpoint of interest. Users can simply supply FuSpot with alignment data from the chimeric aligner together with the breakpoint reported by their detector of choice. In return, FuSpot will retrieve the flanking references, extract reads local to the breakpoint, and construct a cogent representation of the candidate fusion using all the evidence embedded in the sequencing data. Thus, FuSpot allows researchers to thoroughly investigate any fusion breakpoint even if the detection tool they use does not provide sufficient information to do so alone.

### Additional applications

In addition to being a companion tool to visually examine fusion candidates from existing fusion detectors, the flexible nature of FuSpot is designed with the future in mind. Recently, we came across a special type of gene fusion in the form of circular RNA, implicated by some to be a new cancer therapy target [29]. Circular RNA results when the 3′ end of a gene loops and fuses to a preceding 5′ end. Current fusion detectors may miss this type of fusion due to a lack of targeted, specialized filtering. Researchers would then be forced to loosely align their reads with a chimeric aligner and filter for circular style breakpoints unaided by any tool. With the availability of FuSpot, once reads are aligned and candidate breakpoints are identified by the researcher, researchers can then use FuSpot to extract all reads local to the breakpoint, gather the novel breakpoint references and analyze this unique form of fusion meaningfully. We will be watchful in gauging the utility of FuSpot as scientists continue to explore the genome in finer granularity.

## Conclusions

By presenting fusion data in a visually pleasing and intuitive manner, FuSpot puts the analytical power in the hands of the researcher, rather than the algorithm. Our tool empowers researchers to work with even the most sensitive fusion detectors by allowing them to easily identify and eliminate obvious false positives and to systematically select candidates for downstream validation. By enabling quick visualization of real-world fusion candidates, we hope to facilitate gene fusion studies that will lead to more targeted cancer therapies.

## Availability and requirements

Project name: FuSpot

Project home page: http://bioserv.mps.ohio-state.edu/FuSpot

Archived version: Not Applicable

Operating system(s): Web application, Platform independent

Programming language: Python, PHP, JavaScript, HTML, CSS

Other requirements:

- FuSpot website: Modern web browser
- Read extraction helper script: python2.7

License: GPL-3.0

Any restrictions to use by non-academics: None

## Abbreviations

BAM: Binary Alignment Map
BEERS: Benchmarker for Evaluating the Effectiveness of RNA-Seq Software (RNA-Seq data set simulator)
Bp: base pair (used as a unit of measurement)
DAS: Direct Attached Storage
DNA: Deoxyribonucleic Acid
Kb: kilobase pair (a unit of measurement equal to 1000 base pairs of DNA or RNA)
mRNA: messenger Ribonucleic Acid
mRNA-Seq: messenger Ribonucleic Acid Sequencing
NCBI: National Center for Biotechnology Information
PCR: Polymerase Chain Reaction
RNA: Ribonucleic Acid
RNA-Seq: Ribonucleic Acid Sequencing
SRA: Sequence Read Archive
STAR: Spliced Transcripts Alignment to a Reference (RNA Aligner software)
UCSC: University of California, Santa Cruz
